# Regulation of Aldo-keto-reductase family 1 B10 by 14-3-3ε and their prognostic impact of hepatocellular carcinoma

**DOI:** 10.18632/oncotarget.5734

**Published:** 2015-10-19

**Authors:** Tzu-An Liu, Yee-Jee Jan, Bor-Sheng Ko, Yi-Ju Wu, Yi-Jhu Lu, Shu-Man Liang, Chia-Chia Liu, Shyh-Chang Chen, John Wang, Song-Kun Shyue, Jun-Yang Liou

**Affiliations:** ^1^ Institute of Cellular and System Medicine, National Health Research Institutes, Zhunan 350, Taiwan; ^2^ Department of Pathology and Laboratory Medicine, Taichung Veterans General Hospital, Taichung 407, Taiwan; ^3^ Department of Internal Medicine, National Taiwan University Hospital, Taipei 100, Taiwan; ^4^ Institute of Molecular Medicine, National Tsing Hua University, Hsinchu 300, Taiwan; ^5^ Institute of Biomedical Sciences, Academia Sinica, Taipei 115, Taiwan; ^6^ Graduate Institute of Basic Medical Science, China Medical University, Taichung 404, Taiwan

**Keywords:** 14-3-3ε, AKR1B10, β-catenin, hepatocellular carcinoma

## Abstract

14-3-3ε is overexpressed in hepatocellular carcinoma (HCC) and its expression significantly associates with a poor prognostic outcome. To uncover how 14-3-3ε contributes to the tumor progression of HCC, we investigated the potential downstream targets regulated by 14-3-3ε. We found that 14-3-3ε increases expression and nuclear translocation of β-catenin and that 14-3-3ε-induced cell proliferation is attenuated by β-catenin silencing in HCC cells. Moreover, 14-3-3ε induces aldo-keto reductase family 1 member B10 (AKR1B10) expression through the activation of β-catenin signaling. Knockdown of AKR1B10 by siRNAs abolished 14-3-3ε-induced *in vitro* cell proliferation, anchorage-independent growth as well as *in vivo* tumor growth. Furthermore, AKR1B10 silencing increased retinoic acid (RA) levels in the serum of tumor-bearing mice and RA treatment attenuated 14-3-3ε-induced HCC cell proliferation. We further examined 14-3-3ε and AKR1B10 expression and clinicopathological characteristics of HCC tumors. Although the expression of AKR1B10 was significantly correlated with 14-3-3ε, an increase of AKR1B10 expression in 14-3-3ε positive patients paradoxically had better overall survival and disease-free survival rates as well as lower metastatic incidence than those without an AKR1B10 increase. Finally, we found a loss of AKR1B10 expression in cells exhibiting a high capacity of invasiveness. Silencing of AKR1B10 resulted in inducing snail and vimentin expression in HCC cells. These results indicate that AKR1B10 may play a dual role during HCC tumor progression. Our results also indicate that 14-3-3ε regulates AKR1B10 expression by activating β-catenin signaling. A combination of 14-3-3ε with AKR1B10 is a potential therapeutic target and novel prognostic biomarker of HCC.

## INTRODUCTION

The 14-3-3 protein family comprises seven isoforms (β, ε, γ, η, σ, τ/θ, and ζ) in all mammals and is implicated in regulating multiple cellular and physiological functions [1–3]. 14-3-3 proteins are involved in regulating tumor progression of various malignancies [4–9]. Some 14-3-3 isoforms, including 14-3-3β, 14-3-3ε, 14-3-3γ, 14-3-3σ and 14-3-3ζ, are overexpressed and associated with poor prognosis and tumor progression in HCC [10–16]. 14-3-3ε is significantly associated with poor survival rates and a higher incidence of distant metastasis in hepatocellular carcinoma (HCC) patients [10]. Elevated expression of 14-3-3ε promotes epithelial-mesenchymal transition (EMT) and cell migration by reducing E-cadherin levels and inducing focal adhesion kinase expression in HCC cells [17, 18]. However, whether 14-3-3ε is involved in modulating HCC tumor growth and advancing tumor progression remains unclear.

Aldo-keto-reductase family 1 member B10 (AKR1B10, human small intestine aldose reductase or aldose reductase-like) is a NADPH-dependent oxidoreductase and is considered a detoxification enzyme [19, 20]. AKR1B10 is overexpressed in distinct types of malignancies, including lung carcinoma, uterine carcinoma, cholangiocarcinomas as well as breast cancer [21–24]. The expression of AKR1B10 is increased in liver cancer [20, 25–30] and associated with chronic hepatitis C [29]. Silencing of AKR1B10 with siRNA suppresses HCC tumor growth [30]. Although the expression of AKR1B10 is increased in primary HCC tumors, several studies indicate that AKR1B10 is paradoxically correlated with the less aggressive and well-differentiated HCC tumors [23, 31–33]. These results suggest that decreased expression of AKR1B10 is associated with the more advanced and malignant HCC. However, the molecular mechanisms of AKR1B10 and its roles in early stage and advanced HCC have not been well addressed. We discovered that AKR1B10 is regulated by 14-3-3ε/β-catenin signaling and that AKR1B10 contributes to 14-3-3ε-induced HCC cell proliferation and tumor growth. Interestingly, HCC patients that are 14-3-3ε positive and have increased AKR1B10 expression in primary tumors have better prognostic outcomes compared to those without any increase in AKR1B10. Finally, we found that expression of AKR1B10 was eliminated in highly invasive HCC cells. Silencing of AKR1B10 induces the expression of snail and vimentin in HCC cells. This suggests that AKR1B10 may play a dual role during HCC tumor progression. Therefore, 14-3-3ε and AKR1B10 are potential prognostic markers and therapeutic targets of HCC.

## RESULTS

### 14-3-3ε promotes cell proliferation of HCC via β-catenin activation

**Figure 1:**
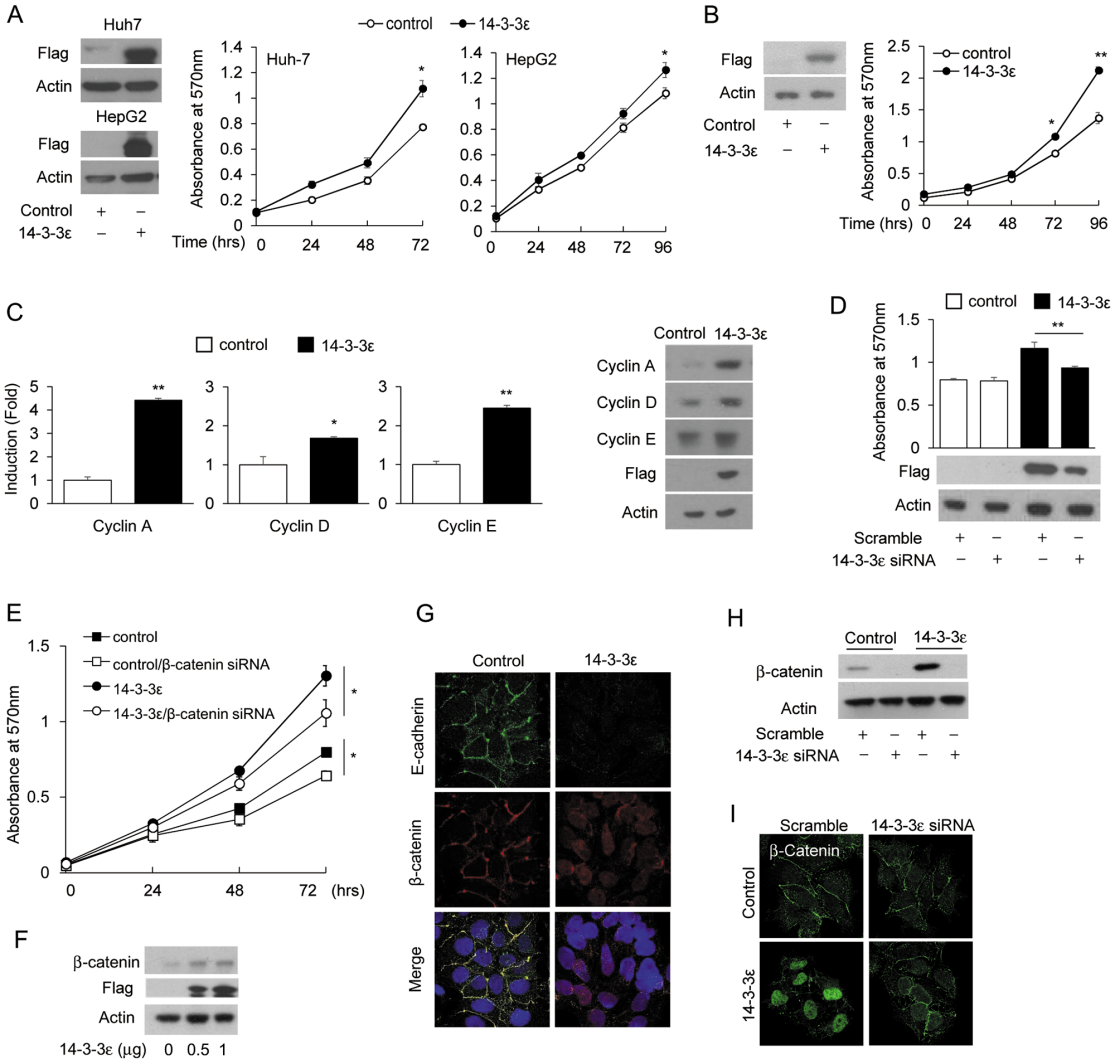
14-3-3ε induces HCC cell proliferation via β-catenin signaling activation **A.** 14-3-3ε was transiently transfected in Huh-7 and HepG2 cells followed by an MTT assay (right panel). The expression of overexpressed 14-3-3ε was confirmed by Western blotting flag analysis (left panel). **B.** Cell proliferation was determined by an MTT assay in 14-3-3ε stably overexpressed (14-3-3ε) and control vector (control) Huh-7 cells (right panel). Expression of overexpressed 14-3-3ε was confirmed by Western blotting flag analysis (left panel). **C.** Cyclin A, cyclin D and cyclin E expressions were determined by RT-PCR (left panel) and Western blotting analysis (right panel) in control and 14-3-3 stable cells. (D, E) 14-3-3ε and β-catenin were knocked down by scramble control and siRNAs in control and 14-3-3ε stable cells for 48 hrs. Cell proliferation was determined by MTT analysis. Overexpressed 14-3-3ε expressions were determined by Western blotting flag analysis (left panel). **F.** Huh-7 cells were transfected with the indicated doses of 14-3-3ε overexpression vectors for 48 hrs. β-catenin and flag expressions were determined by Western blot analysis. **G.** E-cadherin and β-catenin expressions and subcellular localizations were examined by immunofluorescence confocal microscopy in control and 14-3-3ε stable cells. **H.** Control and 14-3-3ε stable cells were transfected with scramble and 14-3-3ε siRNA. β-catenin expression was determined by Western blot analysis. **I.** Control and 14-3-3ε stable cells were transfected with scramble and 14-3-3ε siRNA and subcellular localization of β-catenin was examined by immunofluorescence confocal microscopy. Actin was used as loading control for Western blotting analysis. Scale bars: mean ± SD. *, *P* < 0.05; **, *P* < 0.01.

To investigate whether 14-3-3ε regulates HCC cell proliferation, Huh-7 and HepG2 cells were transiently transfected with a 14-3-3ε overexpression construct (flag-tagged), followed by MTT analysis. Transient overexpression of 14-3-3ε was confirmed by Western blotting analysis of flag-Huh-7 and flag-HepG2 (Figure 1A, left panel). 14-3-3ε induced cell proliferation at 72 hrs was compared to the control vector transfected cells (Figure 1A, right panel). Since we have already established stable cells of Huh-7 for 14-3-3ε overexpression (confirmed by Western blotting analysis, Figure 1B, left panel), we validated the effect on cell proliferation of 14-3-3ε overexpression and found a similar effect that 14-3-3ε enhances cell growth in stable cells (Figure 1B, right panel). 14-3-3ε-induced cell proliferation was further confirmed by the expression of cyclin A, cyclin D and cyclin E, as analyzed by RT-PCR (Figure 1C, left panel) and Western blotting analysis (Figure 1C, right panel). Furthermore, the effect of 14-3-3ε-induced cell proliferation was abrogated by a knockdown experiment with 14-3-3ε siRNA (Figure 1D). These results indicate that increased 14-3-3ε expression results in enhanced HCC cell proliferation.

Wnt/β-catenin signaling has been implicated in promoting tumor progression. We therefore investigated whether β-catenin is involved in 14-3-3ε-induced HCC cell proliferation. The silencing of β-catenin by siRNA significantly reduced cell proliferation in both the control and 14-3-3ε overexpressed cells (Figure 1E). We next studied whether 14-3-3ε alters the expression and activation of β-catenin. Transient overexpression of 14-3-3ε increased β-catenin expression (Figure 1F). Moreover, the result from immunofluorescence staining indicated that 14-3-3ε overexpression reduces E-cadherin expression at the cell junction, thereby increasing nuclear translocation of β-catenin (Figure 1G). In addition, we found that 14-3-3ε siRNA impairs 14-3-3ε-increased β-catenin expression (Figure 1H) and consequently reduces the accumulation of β-catenin in the nuclei by promoting the sequestration of β-catenin at cell junctions in 14-3-3ε-overexpressing cells (Figure 1I). These results indicate that 14-3-3ε promotes HCC cell proliferation *via* β-catenin signaling activation.

### AKR1B10 regulated by 14-3-3ε/β-catenin signaling

**Figure 2:**
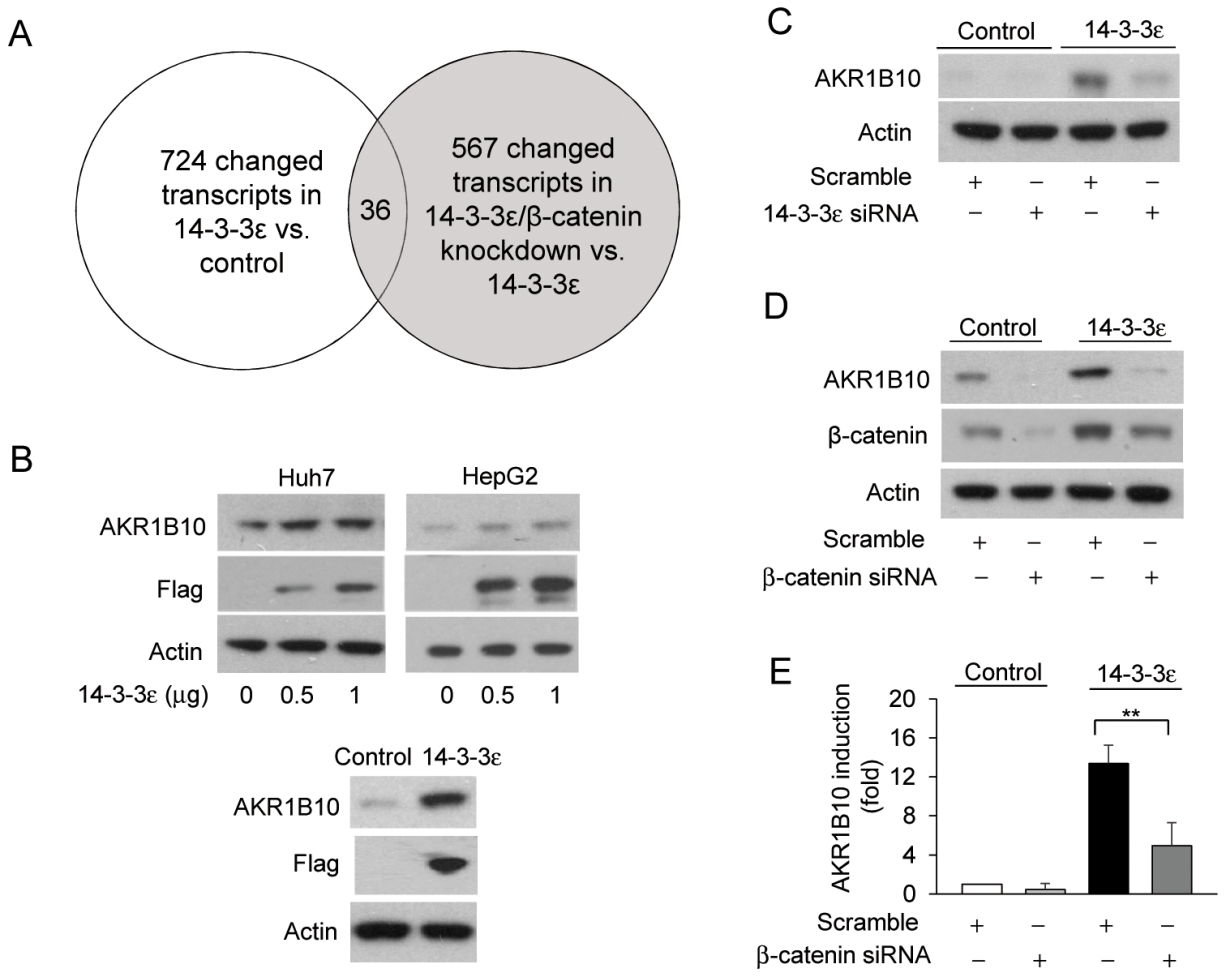
AKR1B10 is regulated by the 14-3-3ε/β-catenin axis **A.** Gene expression profiles for 14-3-3ε overexpression (14-3-3ε stable cells *vs*. control cells) and β-catenin silencing (β-catenin siRNA *vs*. scramble in 14-3-3ε stable cells) were analyzed by microarray analysis. **B.** Upper panel: Huh-7 and HepG2 cells were transiently transfected with the indicated doses of 14-3-3ε overexpression vectors for 48 hrs. AKR1B10 and flag expressions were determined by Western blot analysis. Lower panel: AKR1B10 expression was determined by Western blot analysis in control and 14-3-3ε stable cells. Actin was used as loading control. **C–E.** Control and 14-3-3ε stable cells were transfected with scramble, 14-3-3ε and β-catenin siRNAs as indicated. AKR1B10 and β-catenin expressions were determined by Western blot analysis and RT-PCR analysis. Scale bars: mean ± SD. *, *P* < 0.05; **, *P* < 0.01.

To further investigate how 14-3-3ε/β-catenin signaling alters HCC cell proliferation, we studied the gene expression profile *via* microarray analysis. A total of 724 transcripts were changed by 14-3-3ε overexpression and 567 transcripts were regulated by β-catenin siRNA compared to their controls. There are 36 genes (Supplementary Tables S3 and S4) that overlap between these two groups. These genes are potential downstream targets of 14-3-3ε/β-catenin signaling (Figure 2A). Among these genes, AKRR1B10 was reported to be overexpressed in HCC tumors.

We next validated whether AKR1B10 is regulated by 14-3-3ε/β-catenin signaling. We first confirmed that 14-3-3ε overexpression increases AKR1B10 expression in both transient and stable 14-3-3ε overexpressing cells (Figure 2B, upper panel: transient transfected cells; lower panel: stable cells). Knockdown of 14-3-3ε and β-catenin by siRNA significantly attenuated 14-3-3ε-induced AKR1B10 expression (Figure 2C and 2D). The impairment of AKR1B10 expression by β-catenin siRNA was validated by RT-PCR (Figure 2E).

### AKR1B10 involved in 14-3-3ε-induced cell proliferation and tumor growth

**Figure 3:**
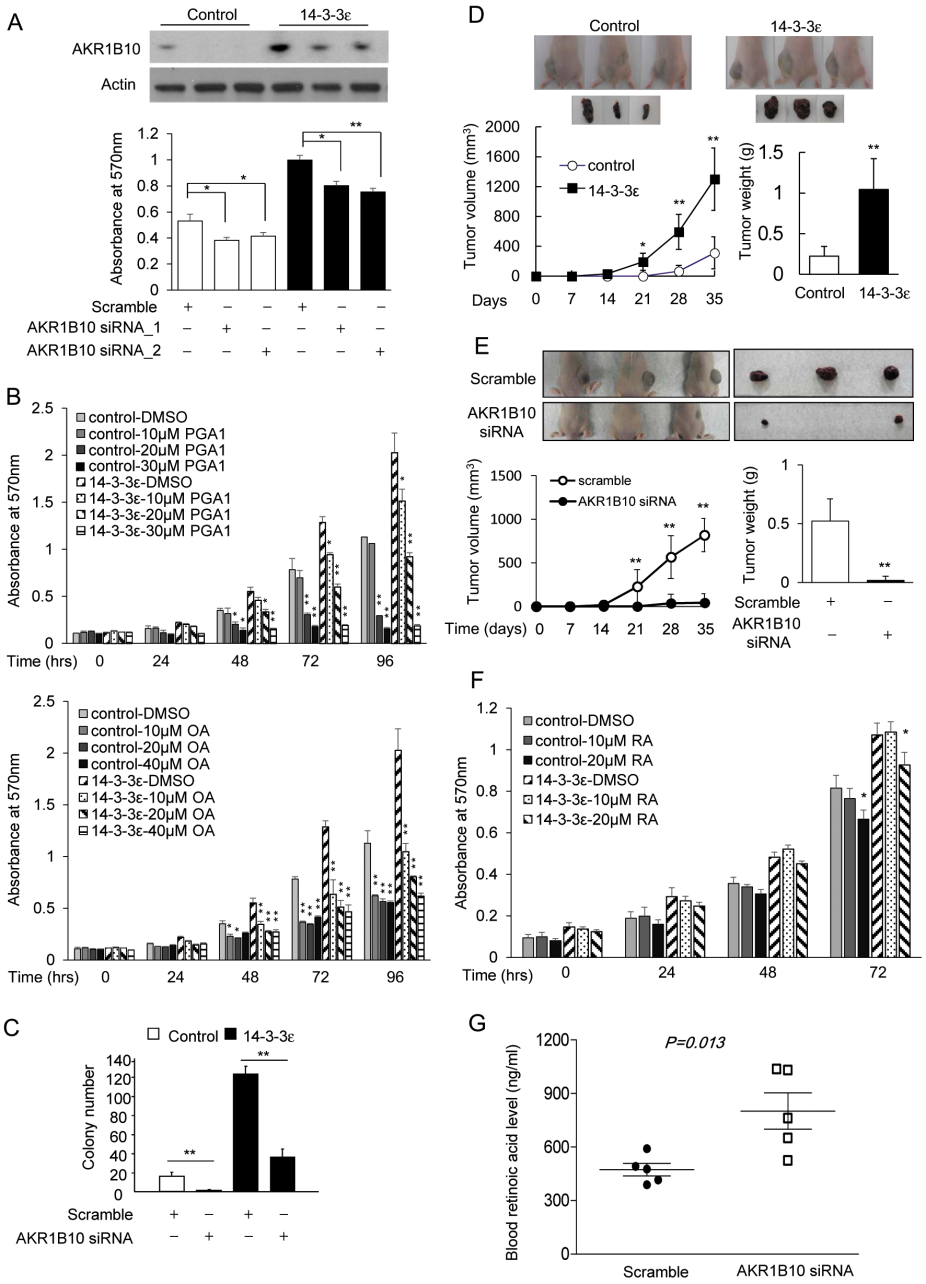
AKR1B10 contributes to 14-3-3ε-induced cell proliferation and tumor growth **A.** Control and 14-3-3ε stable cells were transfected with scramble and two sequences of AKR1B10 siRNAs. AKR1B10 expression was determined by Western blot analysis and cell proliferation was determined by an MTT assay. Actin was used as loading control. **B.** Control and 14-3-3ε stable cells were treated with different concentrations of PGA1 and OA as indicated. Cell proliferation was analyzed by an MTT assay. **C.** Control and 14-3-3ε stable cells were transfected with scramble and AKR1B10 siRNAs. The colony formation number was determined by an anchorage-independent growth assay. **D.** Representative photographs of tumors developed in nude mice injected with control and 14-3-3ε stable cells (upper panel) at Day 35. Tumor growth was examined by calculating the weekly tumor volume (lower panel, left) and tumor weight as determined at day 35 (lower panel, right). *N* = 6, scale bars: mean ± SD. *, *P* < 0.05; **, *P* < 0.01. **E.** 14-3-3ε stable cells were transfected with scramble and AKR1B10 siRNA for 48 hrs. Nude mice were injected with transfected cells for 5 weeks. Tumor growths were examined by calculating the weekly tumor volume (lower panel, left) and tumor weight as determined at day 35 (lower panel, right). *N* = 8, scale bars: mean ± SD. *, *P* < 0.05; **, *P* < 0.01. **F.** Control and 14-3-3ε stable cells were treated with different concentrations of RA for 72 hrs. Cell proliferation was determined by an MTT assay. **G.** Sera were harvested from scarified mice injected with scramble and AKR1B10 siRNA. The RA level in sera of mice was measured by a human retinoic acid ELISA kit. Scale bars: mean ± SD. *, *P* < 0.05; **, *P* < 0.01.

We next studied whether 14-3-3ε-induced AKR1B10 expression is involved in regulating HCC cell proliferation and tumor growth. Silencing of AKR1B10 was performed by siRNA transfection. We found that AKR1B10 siRNA attenuates 14-3-3ε-induced HCC cell proliferation (Figure 3A). To further confirm the role of AKR1B10 in promoting HCC cell proliferation, control and 14-3-3ε overexpressing cells were treated with different concentrations of AKR1B10 inhibitors, PGA1 [34] and OA [35]. PGA1 and OA suppressed cell proliferation in a concentration-dependent manner (Figure 3B). In addition, the effect of AKR1B10 on promoting HCC cell growth was determined by an anchorage-independent cell growth assay. AKR1B10 siRNA significantly suppressed 14-3-3ε-induced HCC cell growth (Figure 3C).

Additionally, an *in vivo* study was performed to elucidate the effect of 14-3-3ε/AKR1B10 expression on HCC tumor growth. 14-3-3ε stable and control cells were subcutaneously injected into nude mice and tumor volume was examined every week. The tumor weight was determined at the end point after sacrificing the mice at day 35. Overexpression of 14-3-3ε significantly promoted tumor growth and increased tumor volume and weight (Figure 3D). 14-3-3ε overexpressing cells were then transfected with AKR1B10 and scramble siRNAs, and subsequently injected into nude mice. AKR1B10 knockdown significantly suppressed 14-3-3ε-induced HCC tumor growth, tumor volume and tumor weight (Figure 3E). These results suggest that AKR1B10 is involved in regulating 14-3-3ε-induced HCC cell proliferation and tumor growth.

AKR1B10 was reported to function as a crucial enzyme in regulating vitamin A metabolism [36–38]. AKR1B10 also reduces RA levels and RA has been implicated in inhibiting cancer cell proliferation [36–38]. Given these facts, we thus hypothesized that 14-3-3ε-induced AKR1B10 may promote HCC tumor growth *via* regulation of RA production. Control and 14-3-3ε overexpressing cells were treated with different concentrations of RA and cell proliferation was determined by an MTT assay. Treatment of RA attenuated 14-3-3ε-induced cell proliferation (Figure 3F). Moreover, we determined the RA level in sera of tumor-bearing mice for control and AKR1B10 siRNA groups mentioned in Figure 3E. The RA level in sera of mice with injected AKR1B10 knockdown cells were significantly higher than in the control group (Figure 3G). These results suggest AKR1B10 may promote HCC tumor growth by reducing RA production.

### 14-3-3ε and AKR1B10 expression correlations and associations with impacts on HCC prognosis

**Figure 4:**
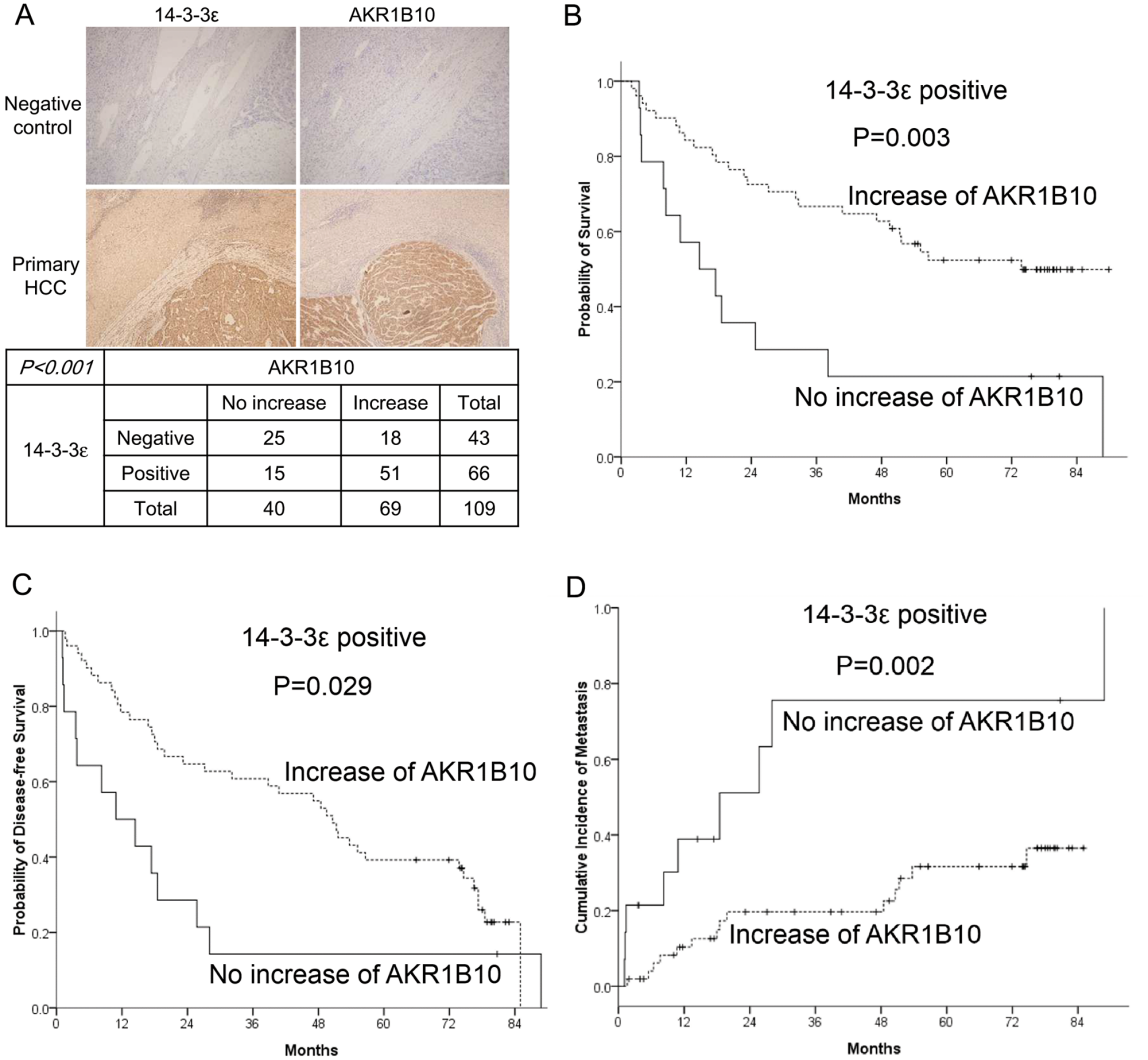
Prognostic analysis of 14-3-3ε and AKR1B10 in HCC tumors **A.** Representative expressions of 14-3-3ε and AKR1B10 in primary tissues of HCC were examined by immunohistochemical analysis (upper panel). Significant correlation of 14-3-3ε and AKR1B10 expression in primary HCC tumors was analyzed using the Chi-square test (lower panel). **B.** A Kaplan-Meier analysis indicates that increased levels of AKR1B10 confer a better overall survival (*P* = 0.003) and **C.** disease-free survival (*P* = 0.029) as well as **D.** a lower metastatic risk (*P* = 0.002) when compared with no increase of AKR1B10 in 14-3-3ε positive patients.

We have previously shown that 14-3-3ε is overexpressed in primary HCC tumors and significantly associated with an increased risk of extrahepatic metastasis and reduced overall survival [10]. To evaluate whether AKR1B10 is a crucial downstream effector of 14-3-3ε in regulating HCC progression, the expressions of AKR1B10 and 14-3-3ε were analyzed by immunohistochemistry (IHC) in a retrospective cohort of 109 HCC tissues. AKR1B10 expression was tightly correlated with 14-3-3ε in HCC tumors, as determined by IHC staining (Figure 4A). In addition to a positive correlation with 14-3-3ε (*p* < 0.001), the expression of AKR1B10 was compared with other clinicopathological characteristics. AKR1B10 expression exhibited an association with free surgical margins (*p* = 0.011), early BCLC staging (*p* = 0.042) and a lack of subsequent extrahepatic metastasis (*p* = 0.047) (Table 1). Multi-variate analysis of potential prognostic factors for overall survival, disease-free survival and metastasis was accomplished by means of COX hazard regression (Table 2). AKR1B10 expression significantly associated with overall survival (*p* = 0.015) and metastasis (*p* = 0.011) in 14-3-3ε positive HCC patients (Table 2).

**Table 1:** Correlation of AKR1B10 expression with 14-3-3ε and clinicopathological characteristics in primary tumors of HCC patients

Parameters	Increase of AKR (Q-score increase ≥ 2) % (*n*)	*p*-value
Overall (*n* = 109)	63.3% (69)	
Age ≥ 60 years (*n* = 54) < 60 years (*n* = 55)	64.8% (35) 61.8% (34)	NS
Gender Male (*n* = 82) Female (*n* = 27)	62.2% (51) 66.7% (18)	NS
Histology grade 1 (*n* = 7) 2 (*n* = 78) 3 (*n* = 24)	42.9% (3) 66.7% (52) 58.3% (14)	NS
Types of surgery Wedge resection (*n* = 39) Segmentectomy (*n* = 53) Lobectomy (*n* = 17)	69.2% (27) 60.4% (32) 58.8% (10)	NS
Surgical margin Free (*n* = 83) Involved (*n* = 26)	70.0% (58) 42.3% (11)	0.011*
BCLC staging Not available (*n* = 3) Early (stage A1 to A4) (*n* = 56) Intermediate (stage B) (*n* = 48) Advanced (stage C) (*n* = 2)	71.4% (40) 52.1% (25) 50.0% (1)	0.042*
Tumor Size ≥ 5.0 cm (*n* = 34) < 5.0 cm (*n* = 75)	52.9% (18) 68.0% (51)	NS
Tumor multiplicity Single (*n* = 84) Multiple (*n* = 25)	66.7% (56) 52.0% (13)	NS
Capsular formation Not available (*n* = 8) Yes (*n* = 58) No (*n* = 43)	60.3% (35) 60.5% (26)	NS
Micro-vascular thrombi Yes (*n* = 47) No (*n* = 62)	55.3% (26) 69.4% (43)	NS
Liver cirrhosis Not available (*n* = 3) Yes (*n* = 56) No (*n* = 50)	71.4% (40) 54.0% (27)	0.063
Viral Hepatitis Not available (*n* = 7) Hepatitis B (*n* = 54) Hepatitis C (*n* = 30) Both (*n* = 15) None (*n* = 3)	59.3% (32) 53.3% (16) 80.0% (12) 100.0% (3)	NS
Alpha-fetoprotein level Not available (*n* = 12) ≥ 80 ng/ml (*n* = 35) < 80 ng/ml (*n* = 62)	65.7% (23) 59.7% (37)	NS
Subsequent extrahepatic metastasis Yes (*n* = 29) No (*n* = 80)	48.3% (14) 68.8% (55)	0.047*
14-3-3ε positivity Yes (*n* = 66) No (*n* = 43)	77.3% (51) 41.9% (18)	< 0.001*

BCLC, Barcelona-Clinic Liver Cancer; NS, not significant; Q-score, Quick-score

* *p* < 0.05

**Table 2:** Multi-variate analysis by Cox hazard regression for overall survival, disease-free survival and metastasis in 14-3-3ε positive HCC patients

Variable	Overall survival		Disease-free survival		Metastasis	
	Hazard ratio (95% confidence interval)	*p*-value	Hazard ratio (95% confidence interval)	*p*-value	Hazard ratio (95% confidence interval)	*p*-value
Age (≧ 60 vs. < 60 y/o)	1.532 (0.729–3.218)	0.260	0.819 (0.421–1.592)	0.556	0.818 (0.315–2.123)	0.680
Sex (Female vs. male)	0.509 (0.198–1.306)	0.160	1.137 (0.555–2.328)	0.726	1.564 (0.546–4.479)	0.404
BCLC stage (Early vs. Intermediate/late)	0.753 (0.359–1.580)	0.453	0.652 (0.301–1.414)	0.279	0.402 (0.151–1.070)	0.068
AFP level (≧ 80 vs. < 80 ng/ml)	1.309 (0.589–2.910)	0.509	1.141 (0.544–2.393)	0.727	0.519 (0.148–1.826)	0.307
Increased AKR1B10 (Yes vs. No)	0.376 (0.171–0.827)	0.015*	0.646 (0.340–1.227)	0.182	0.262 (0.097–0.731)	0.011*

* *p* < 0.05

To further elucidate the clinical impacts of 14-3-3ε/AKR1B10 expressions on the prognosis of HCC, we examined their expression associations regarding the risk of metastasis and the survival rate. Although AKR1B10 was positively correlated with 14-3-3ε expression (*p* < 0.001), 14-3-3ε positive HCC patients with increased AKR1B10 expression paradoxically had better overall survival (Figure 4B) and progression-free survival (Figure 4C) rates as well as lower metastatic risks (Figure 4D). These results suggest a potential dual effect of AKR1B10 in HCC tumor progression.

### Potential role of AKR1B10 in EMT of HCC

**Figure 5:**
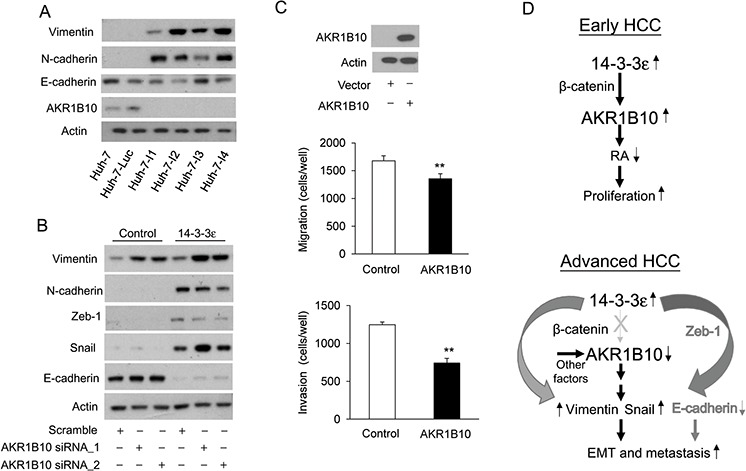
AKR1B10 is involved in regulating EMT Vimentin, N-cadherin, E-cadherin and AKR1B10 expressions were determined by Western blot analysis in Huh-7, Huh-7-Luc, and highly invasive (Huh-7-I1, Huh-7-I2, Huh-7-I3 and Huh-7-I4) cells. Actin was used as a loading control. **B.** Control and 14-3-3ε stable cells were transfected with scramble and two sequences of AKR1B10 siRNAs for 48 hrs. Vimentin, N-cadherin, Zeb-1, snail and E-cadherin expressions were determined by Western blot analysis. Actin was used as a loading control. **C.** Huh-7-I1 cells were transfected with control and AKR1B10 overexpression vectors for 48 hrs. AKR1B10 expression was examined by Western blotting analysis and actin was used as a loading control (upper panel). Effect of AKR1B10 overexpression on cell migration (middle panel) and invasion (lower panel) was determined by Boyden chamber assay. Scale bars: mean ± SD. **, *P* < 0.01. **D.** An illustrated scheme for potential roles of 14-3-3ε and AKR1B10 in regulating HCC tumor progression.

In this study, we found that AKR1B10 plays an important role in 14-3-3ε-induced cell proliferation and HCC tumor growth. Increased AKR1B10 expression was significantly associated with better prognostic outcomes of HCC, whereas lack of AKR1B10 expression represented the worst HCC prognostic outcomes. We therefore postulated that AKR1B10 is involved in regulating EMT and the consequent distant HCC metastases. To test this hypothesis, we selected highly invasive HCC cells from Huh-7 cells using the two-chamber system. These highly invasive cells were established using multiple selections and confirmed by an *in vitro* invasion assay. Expressions of EMT markers, including vimentin, N-cadherin and E-cadherin, in control cells (parental Huh-7 and luciferase transfected Huh-7, Huh-7-Luc) and highly invasive cells (Huh-7-I1, Huh-7-I2, Huh-7-I3 and Huh-7-I4) were determined by Western blot analysis. The highly invasive cells significantly expressed higher amounts of vimentin and N-cadherin, but there was slightly decreased E-cadherin expression (Figure 5A). Interestingly, a loss of AKR1B10 expression was observed in these highly invasive cells, showing a negative correlation with EMT marker expressions (Figure 5A).

To further investigate whether AKR1B10 modulates EMT, AKR1B10 was knocked down by siRNA in control and 14-3-3ε overexpressing cells. Knockdown of AKR1B10 induced vimentin and snail expression but only slightly reduced N-cadherin and Zeb-1 and increased E-cadherin (Figure 5B).

We next performed experiments to determine the effect of AKR1B10 on cell migration and invasion *via* a Boyden chamber assay. Highly invasive (Huh-7-I1) cells were transiently transfected with AKR1B10 overexpression vectors for 48 hrs. The expression of overexpressed AKR1B10 was confirmed by Western blotting analysis (Figure 5C, upper panel). AKR1B10 overexpression suppressed cell migration (Figure 5C, middle panel) and invasion (Figure 5C, lower panel) of Huh-7-I1 cells. These results suggest that reductions in AKR1B10 expression may enhance EMT and subsequent metastasis in advanced HCC.

## DISCUSSION

β-catenin is a crucial downstream effector of Wnt signaling [39, 40]. β-catenin stability is regulated by proteasome degradation, and β-catenin interacts with cadherin proteins to maintain tight junction cellular functions [39, 40]. A previous study reported that β-catenin expression is involved in promoting tumor growth and associated with poorer survival [41] and the Wnt/β-catenin pathway is potentially linked to the transforming growth factor-β (TGF-β) signaling of HCC [42]. Our previous study indicated that 14-3-3ε suppresses E-cadherin expression *via* the induction of Zeb-1 expression [17]. In this study, we demonstrated that 14-3-3ε not only induces the β-catenin expression level (Figure 1F), but also promotes nuclear translocation of β-catenin (Figure 1G). Another study reported that overexpression of selective 14-3-3σ isoforms enhances mouse embryonic stem cell proliferation by sequestering GSK-3β and stabilizing β-catenin [43]. It is possible that β-catenin stability and activity are altered by 14-3-3ε in HCC due to isoform and tissue specificity. Thus, our results provide support that 14-3-3ε promotes HCC tumor progression by modulating β-catenin signaling. Moreover, we have shown for the first time that AKR1B10 is regulated by β-catenin (Figure 2D and 2E). However, whether the β-catenin/Tcf/Lef complex directly binds to the AKR1B10 promoter region needs further investigation.

In addition to AKR1B10, we found that 14-3-3ε upregulates AKR1C3 expression through β-catenin-dependent signaling according to the results of the microarray analysis (Supplementary Table S3). As AKR1C3 is highly expressed in some human cancers, including HCC [44], the transcriptional regulation of certain isoforms of the AKR family may be mediated by similar mechanisms.

Interleukin 18 (IL-18) is one of the potential targets upregulated by 14-3-3ε/β-catenin signaling (Supplementary Table S3). IL-18 in serum has been reported as a potential prognostic factor of HCC [45] and it may be the downstream effector of HCC progression promoted by 14-3-3ε. In addition, vimentin is thought to contribute to EMT and tumor metastasis. Our previous study indicated that 14-3-3ε induces vimentin expression [17]. We currently found that this expression is abolished by β-catenin silencing (Supplementary Table S3). Vimentin is thus another potential downstream factor regulated by 14-3-3ε/β-catenin signaling.

Upregulation of AKR1B10 results in depletion of RA [36–38]. RA exhibits profound effects on modulating cell differentiation, maturation and proliferation [46, 47]. We hypothesized that elevated 14-3-3ε expression may reduce RA levels by AKR1B10 induction. Both AKR1B10 inhibitors PGA1 and OA suppressed HCC cell proliferation (Figure 3B). Interestingly, we found that PGA1 induced AKR1B10 expression whereas OA had no effect on AKR1B10 (Figure Supplementary S1). These results imply that these inhibitors may not be selective for inhibitory activity of AKR1B10. We therefore employed a siRNA silencing approach to validate the effect of AKR1B10 on cell proliferation (Figure 3C). We further found that PGA1 and OA are more effective than RA in suppressing cell proliferation (Figure 3B *vs*. 3F). This suggests that other potential downstream effectors of AKR1B10 are possibly involved. Moreover, we found RA induced AKR1B10 expression in 14-3-3ε overexpressing cells (Figure Supplementary S2). Since AKR1B10 may attenuate RA production, this suggests that there may be negative feedback machinery between AKR1B10 and RA in 14-3-3ε overexpressed HCC cells. Further investigation is needed to elucidate this complicated association.

We determined the level of RA in the sera of mice that were injected with 14-3-3ε overexpressing cells and compared those levels with the control cells. We found RA levels in mice with 14-3-3ε overexpressing cells to be relatively higher than the control group although the statistics were not significant. This may due to insufficient sample numbers (Figure Supplementary S3). However, silencing of AKR1B10 expression in 14-3-3ε overexpressing cells resulted in an increase in the levels of retinoic acid (Figure 3G) in the tumor-bearing mice, which was significantly correlated with reduction of tumor size (Figure 3E). These results suggest RA may be involved in modulating 14-3-3ε/AKR1B10-induced HCC tumor growth.

Moreover, AKR1B10 has been reported to promote cell survival by regulating lipid synthesis and eliminating production of carbonyls [48]. Silencing of AKR1B10 significantly increased unsaturated carbonyls and induced cellular lipid peroxides [48]. It is therefore a reasonable speculation that AKR1B10 contributes to tumor growth by reducing RA production and regulating lipid metabolism. Alterations of the lipid synthetic profile may contribute to the tumor microenvironment, thereby modulating tumor progression. AKR1B10 is a secreted factor and can potentially serve as a prognostic serum marker for breast cancer [24]. Interestingly, 14-3-3 proteins have been found in cell-secreted exosomes and suggested to contribute to the tumor associated microenvironment [49, 50]. Thus, 14-3-3ε may synergize with AKR1B10 to promote tumor growth, not only by autocrine regulation in cancer cells, but also in regulating tumor associated stromal cells in a paracrine manner. Whether the 14-3-3ε/β-catenin/AKR1B10 axis is involved in the tumor microenvironment or in “educating” cancer associated stromal cells by affecting lipid metabolism needs to be further investigated.

The role of AKR1B10 during HCC tumor prognosis is paradoxical. AKR1B10 is overexpressed in HCC and the silencing of AKR1B10 suppresses HCC cell proliferation [30]. Conversely, the positive expression of AKR1B10 associates with early HCC, suggesting AKR1B10 is a potential marker of early HCC [31–33]. Our results indicate that 14-3-3ε-induced AKR1B10 promotes HCC cell proliferation and tumor growth. However, we found that patients with an increase in AKR1B10 have better clinical outcomes than those without an increase of AKR1B10 in 14-3-3ε positive patients (Figure 4B–4D). Interestingly, although AKR1B10 promotes cell proliferation, we found that the expression of AKR1B10 was almost undetectable in HCC cells with high invasive capacity (Figure 5A).

Overexpression of AKR1B10 suppresses cell migration and invasion of highly invasive cells (Figure 5C). We further investigated this by examining the incidence rate of tumor formation of these invasive cells *in vivo* in a nude mice model. Although the tumors were initiated within two weeks after injection of control (Figure 3D) or Huh-7-Luc cells (data not show), highly invasive cells lost the capacity for tumor formation (Figure Supplementary S4). Moreover, silencing of AKR1B10 significantly induced EMT markers of vimentin and snail expression but had only a slight effect on Zeb-1, E-cadherin and N-cadherin (Figure 5B). These results suggest the regulation of HCC tumor progression by the 14-3-3ε/AKR1B10 axis is complicated. AKR1B10 may synergize with other regulators to play paradoxical roles in regulating HCC tumor growth, EMT and metastasis. Moreover, in addition to regulating AKR1B10 expression, 14-3-3ε increases vimentin expression through activation of β-catenin (Supplementary Table S3) and suppresses E-cadherin expression by induction of Zeb-1 [17]. Thus, AKR1B10 may synergize with 14-3-3ε and contribute to modulation of EMT and subsequent metastasis (Figure 5C). Additionally, our study suggests that 14-3-3ε-induced AKR1B10 promotes cancer cell proliferation by reducing RA production in the early stages of primary HCC (Figure 5D, upper panel). The machinery of 14-3-3ε/β-catenin-induced AKR1B10 expression is potentially eliminated in advanced HCC and negative regulation of AKR1B10 by other factors results in enhancing snail/vimentin expression and consequent EMT as well as distant metastasis (Figure 5D, lower panel). AKR1B10 can therefore be thought of as playing dual roles during tumor progression of HCC. Our results regarding the paradoxical effects of AKR1B10 echo the previously reported hypothesis that reduced proliferation and enhanced migration are two sides of the same coin [51].

In this study, we provide evidences that AKR1B10 is upregulated by 14-3-3ε mediated by a β-catenin-dependent mechanism. We found that the decrease in RA production may be involved in AKR1B10-induced HCC cell proliferation and tumor growth. We also demonstrated a paradoxical effect of AKR1B10 for regulation of cell proliferation and EMT. We conclude that the combination of 14-3-3ε with AKR1B10 can be potentially used as a prognostic biomarker and that differentially targeting the 14-3-3ε/AKR1B10 axis is a potential therapeutic strategy for HCC treatment.

## MATERIALS AND METHODS

### Chemicals, cell culture and transfection

Prostaglandin A1 (PGA1), oleanolic acid (OA) and trans-retinoic acid (RA) were purchased from Sigma-Aldrich Chemical Company (St. Louis, MO, USA). The Human Retinoic Acid ELISA Kit was from http://Mybiosource.com (San Diego, CA, USA). Huh-7 and HepG2 human cells were maintained in DMEM (Gibco, Gaithersburg, MD, USA) supplemented with 10% fetal bovine serum (FBS; Hyclone Thermo Fisher Scientific, Waltham, MA, USA), 100 units/ml penicillin, and 100 units/ml streptomycin, in a humidified incubator with 5% CO_2_ at 37°C.

The vector construction of the 14-3-3ε overexpression vector, the transient transfection procedure and the selection of 14-3-3ε stable cells were all carried out as previously described [17]. To establish stable cells, Huh-7 cells were transfected with p3XFlag-CMV (control) and 14-3-3ε overexpression (14-3-3ε) plasmids using the Polyjet™ transfection reagent (SignaGen Laboratories, USA). The transfected cells were screened with G418 (500 μg/ml) for 4 weeks. Single colonies of stable clones were selected and maintained in DMEM with 10% FBS and 200 μg/ml of G418. The established stable cell lines were confirmed by Western blot analysis using the flag expression system. For AKR1B10 overexpression, cDNA of AKR1B10 was amplified and subcloned into pcDNA3.1. Overexpression of AKR1B10 was performed according to the procedure described above.

The selection of highly invasive Huh-7 cells and the invasion assay were performed in a modified Boyden chamber using a cell culture insert (Becton Dickinson, Pont-de-Claix, France) coated with 0.1% matrigel containing 0.1% bovine serum albumin (BSA) in DMEM medium. Huh-7 cells were incubated in the upper chamber and cells with invasive capacity were harvested from the bottom chamber which contained 100 μg/mL fibronectin (Becton Dickinson), 20 ng/ml epidermal growth factor (EGF) and 10% BSA. This two-chamber selection was performed multiple times to establish highly invasive HCC cells.

### Real-time polymerase chain reaction assay

RNA was extracted and prepared using TRIzol (Invitrogen, Grand Island, NY, USA) and cDNA was synthesized by the SuperScript III First-Strand Synthesis Supermix Kit (Invitrogen). RNA expression was quantified using quantitative real-time PCR (RT-PCR). RT-PCR of cyclin A, cyclin D, cyclin E and AKR1B10 was performed using SYBR Green (Kapa Biosystem, Woburn, MA, USA) with specific oligonucleotide primers (Supplementary Table S1) and analyzed by an ABI PRISM 7900 system (Applied Biosystems, Foster City, CA, USA). Gene expression was normalized using actin as an internal control.

### Knockdown studies

Gene silencing was performed by using 14-3-3ε, β-catenin and AKR1B10 siRNAs (Stealth RNAi, Invitrogen, Carlsbad, CA) and scramble control (Invitrogen) with reported sequences (Supplementary Table S2). Transient transfection of siRNA was carried out using Lipofectamine™ RNAiMAX (Invitrogen) according to the manufacturer's guidelines.

### Cell proliferation assay

Cell proliferation was measured by a 3-(4,5-dimethyl thiazol-2-yl)-2,5-diphenyl tetrazolium bromide (MTT) assay, as described previously [12, 17]. Absorbance at 570 nm was measured with a reference wavelength of 690 nm.

### Western blot analysis

Cells were lysed in ice-cold RIPA buffer (Millipore, Temecula, CA, USA) containing cocktail protease inhibitors (Roche, IN, USA). Cell lysates were centrifuged at 15,000 rpm for 20 minutes at 4°C, and protein concentrations were determined. 20 μg of protein from each sample were run through a gradient SDS-PAGE gel, followed by immunoblotting onto PVDF membranes. The membranes were blocked and probed with primary antibodies of actin (Sigma-Aldrich), AKR1B10 and Zeb-1 (Santa Cruz Biotechnologies, Heidelberg, Germany), cyclin A, cyclin D, cyclin E, snail and β-catenin (Cell Signaling Technology, Beverly, MA, USA), E-cadherin and N-cadherin (BD Biosciences, San Jose, CA) and vimentin (Millipore). The membranes were immersed in PBST containing horseradish peroxidase-conjugated secondary antibodies, and protein levels were determined by use of enhanced chemiluminescence reagents.

### Immunofluorescence confocal microscopy

Immunofluorescence staining was performed as described in our previous study [12, 17]. Cells were incubated with the primary antibodies of anti-E-cadherin (BD Biosciences) and anti-β-catenin (Cell Signaling Technology) in PBS containing 1% FBS at 4°C overnight. This was followed by incubation with Alexa Fluor^®^ 488 secondary antibody (Invitrogen) in PBS containing 5% bovine serum albumin at room temperature for 2 hrs. Samples were mounted and images were analyzed using the Leica TCS SP5 Confocal Imaging System (Leica, Germany).

### Microarray analysis

Gene expression regulated by 14-3-3ε overexpression or β-catenin silencing in Huh-7 stable cells was analyzed by microarray analysis. All RNA samples were extracted from control/14-3-3ε overexpressing as well as scramble/β-catenin siRNA transfected cells using Qiagen RNeasy Mini Kit (Qiagen, Valencia, CA, USA) and the microarray analysis was processed according to the manufacturers' instructions (Affymetrix Inc., Santa Clara, CA, USA).

### Anchorage-independent growth assay

Anchorage-independent growth was assessed by a soft agar assay. Briefly, 6 × 10^3^ cells were seeded onto six-well plates with 1 ml DMEM containing 0.8% low-melting agarose (Lonza, Rockland, ME) followed by overlaying with 2 ml DMEM containing 0.8% low-melting agarose. After 3 weeks, cells were stained with 0.005% crystal violet in 25% methanol and colony numbers were counted.

### Tumor xenograft experiments and determination of serum RA level

BALB/c nu/nu nude mice (8 weeks old) were purchased from the National Laboratory Animal Center, National Science Council and housed in micro-isolator cages at a specific pathogen-free facility at the Laboratory Animal Center, National Health Research Institutes of Taiwan. Control and 14-3-3ε stable cells were trypsinized, washed and resuspended in PBS. For AKR1B10 silencing experiments, Huh-7 cells with stable 14-3-3ε overexpression were transfected with scramble or AKR1B10 siRNA for 48 hrs before harvesting the cells. A total of 2 × 10^6^ cells in 0.3 ml PBS w were injected subcutaneously into the right flank of nude mice. Tumor volume was determined by sequential caliper measurements of length (L) and width (W) and calculated as LW^2^/2. After five weeks, mice were scarified, tumors were removed and tumor weight was measured.

For determining serum RA levels, blood from sacrificed mice was collected by heart puncture. The serum was separated by centrifuging the blood for 10 min at 1500 × g and 4°C and storing the serum at − 80°C. The concentration of RA was measured by a human retinoic acid ELISA kit (MYBioSource, San Diego, CA, USA).

### Clinical specimens

109 primary HCC samples were retrospectively obtained from patients who had undergone surgery for tumor resection or biopsy at Taichung Veterans General Hospital from January 1999 to December 2001. Slides from paraffin-embedded surgical specimens of primary tumors with surrounding non-cancerous liver parenchyma were subjected to immunohistochemical (IHC) staining. The pathological features, IHC staining results, clinical parameters, including Barcelona-Clinic liver cancer (BCLC) staging [52], and disease outcomes, were collected for analysis. This study was approved by the Institutional Review Board of Taichung Veterans General Hospital. The policy that states informed consents are not required for using these de-linked samples for retrospective analysis was also approved by the Institutional Review Board.

### Immunohistochemical analysis

For immunohistochemistry analysis, an automatic immunostaining device and an ultraView detection kit (Ventana XT Medical System, Tucson, AZ) were used to detect 14-3-3ε expressions in paraffin-embedded tissues by use of a primary antibody against 14-3-3ε and AKR1B10 (Santa Cruz Biotechnology). A negative control was prepared by the same staining procedure without primary antibodies. The intensity of 14-3-3ε and AKR1B10 protein staining was semiquantitatively scored by a Quick-score (Q-score) method based on intensity and heterogeneity [10–12, 15, 17, 53–55]. Staining intensity was scored as 0 (negative), 1 (weak), 2 (moderate), or 3 (strong). For heterogeneity, the proportions of tumor cells positively stained with 14-3-3ε and AKR1B10 were scored as 0 (0%); 1 (1–25%); 2 (26–50%); 3 (51–75%) or 4 (76–100%). The Q-score of a given tissue sample was the sum of the intensity and heterogeneity scores and ranged from 0 to 7. A Q-score (2 of 14-3-3ε was defined as positive expression, and a Q-score < 2 was considered negative expression. An increase of Q-score (2 for AKR1B10 between cancerous tissues compared with surrounding normal cells was considered increased expression, and an increase with Q-score < 2 or a decrease was considered a lack of increased expression. Cases with < 5% weakly stained specimens were considered as negative expression.

### Migration and invasion assay

Bio-coat cell migration Boyden chamber was used for cell migration assays and invasion assays (with 0.1% matrigel containing 0.1% bovine serum albumin (BSA)-DMEM medium in the upper chambers). Cells were trypsinized, re-suspended and cultured in the upper chambers for 16 h (3 × 10^4^ for migration and 2 × 10^4^ for invasion assay). Cells remaining on the upper side were removed and migrated/invasive cells on the bottom side were fixed as described previously [12, 17]. Cell migration and invasion was quantified by counting the total number of migrated and invasive cells.

### Statistical analysis

The Student's *t*-test was used to analyze differences between two groups. Kaplan-Meier curves were plotted and the log rank test was used to analyze time-related variables of probabilities for metastasis and overall survival. Cox-proportional hazard regression was used in multi-variate analysis for the impact of prognostic factors on overall survival, disease-free survival and metastasis. *P* value < 0.05 was considered statistically significant.

## SUPPLEMENTARY FIGURES AND TABLES


