# Correction: Regulation of Aldo-keto-reductase family 1 B10 by 14-3-3ε and their prognostic impact of hepatocellular carcinoma

**DOI:** 10.18632/oncotarget.26226

**Published:** 2018-10-09

**Authors:** Tzu-An Liu, Yee-Jee Jan, Bor-Sheng Ko, Yi-Ju Wu, Yi-Jhu Lu, Shu-Man Liang, Chia-Chia Liu, Shyh-Chang Chen, John Wang, Song-Kun Shyue, Jun-Yang Liou

**Affiliations:** ^1^ Institute of Cellular and System Medicine, National Health Research Institutes, Zhunan 350, Taiwan; ^2^ Department of Pathology and Laboratory Medicine, Taichung Veterans General Hospital, Taichung 407, Taiwan; ^3^ Department of Internal Medicine, National Taiwan University Hospital, Taipei 100, Taiwan; ^4^ Institute of Molecular Medicine, National Tsing Hua University, Hsinchu 300, Taiwan; ^5^ Institute of Biomedical Sciences, Academia Sinica, Taipei 115, Taiwan; ^6^ Graduate Institute of Basic Medical Science, China Medical University, Taichung 404, Taiwan

**This article has been corrected:** The labelling for equal first authorship is given below:

^*^ These authors contributed equally to this work

Original article: Oncotarget. 2015; 6:38967-38982. https://doi.org/10.18632/oncotarget.5734

