# Supplementary material for: Regulation of Aldo-keto-reductase family 1 B10 by 14-3-3ε and their prognostic impact of hepatocellular carcinoma
Source: Oncotarget. 2015 Oct 19;6(36):38967–82. doi: 10.18632/oncotarget.5734 (PMC4770750; doi:10.18632/oncotarget.5734)
Supplement: Supplementary file 1 [file oncotarget-06-38967-s001.pdf]

SUPPLEMENTARY FIGURES AND TABLES

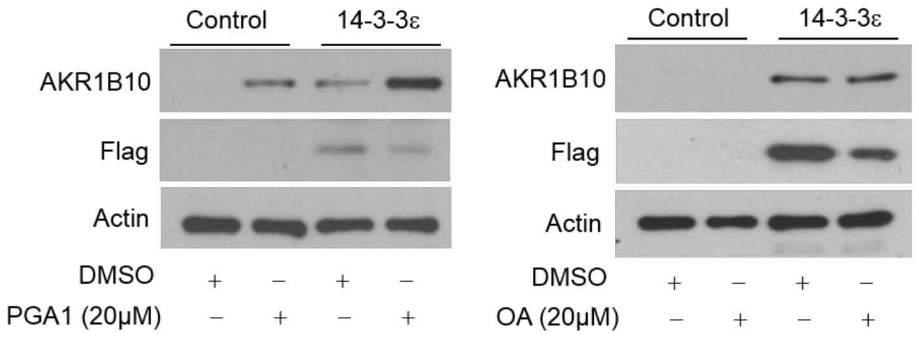

Supplementary Figure S1: Effect of prostaglandin A1 (PGA1) and oleanolic acid (OA) on AKR1B10 expression. Control and 14-3-3ε overexpressed stable cells were treated with PGA1 (left panel) and OA (right panel) for 24 hrs. AKR1B10 and flag expressions were determined by Western blot analysis. Actin was used as loading control.

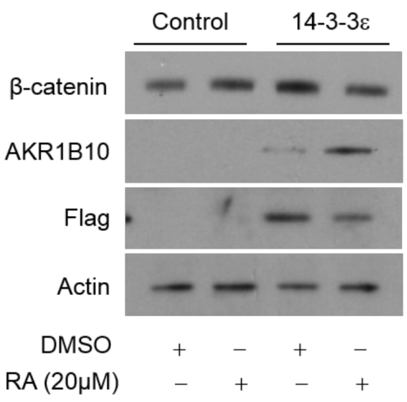

Supplementary Figure S2: Effect of retinoic acid (RA) on AKR1B10 expression. Control and 14-3-3ε overexpressed stable cells were treated with RA for 24 hrs. β-catenin, AKR1B10 and flag expressions were determined by Western blot analysis. Actin was used as loading control.

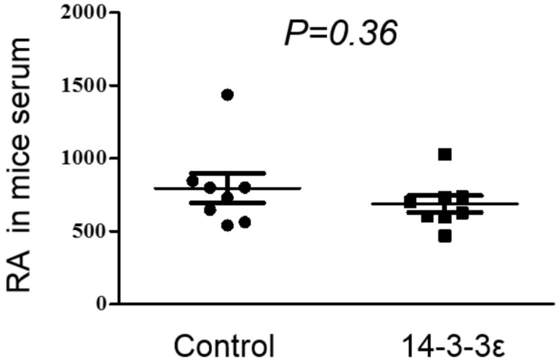

Supplementary Figure S3: Retinoic acid levels in sera from mice injected with control and 14-3-3ε overexpressed stable cells.

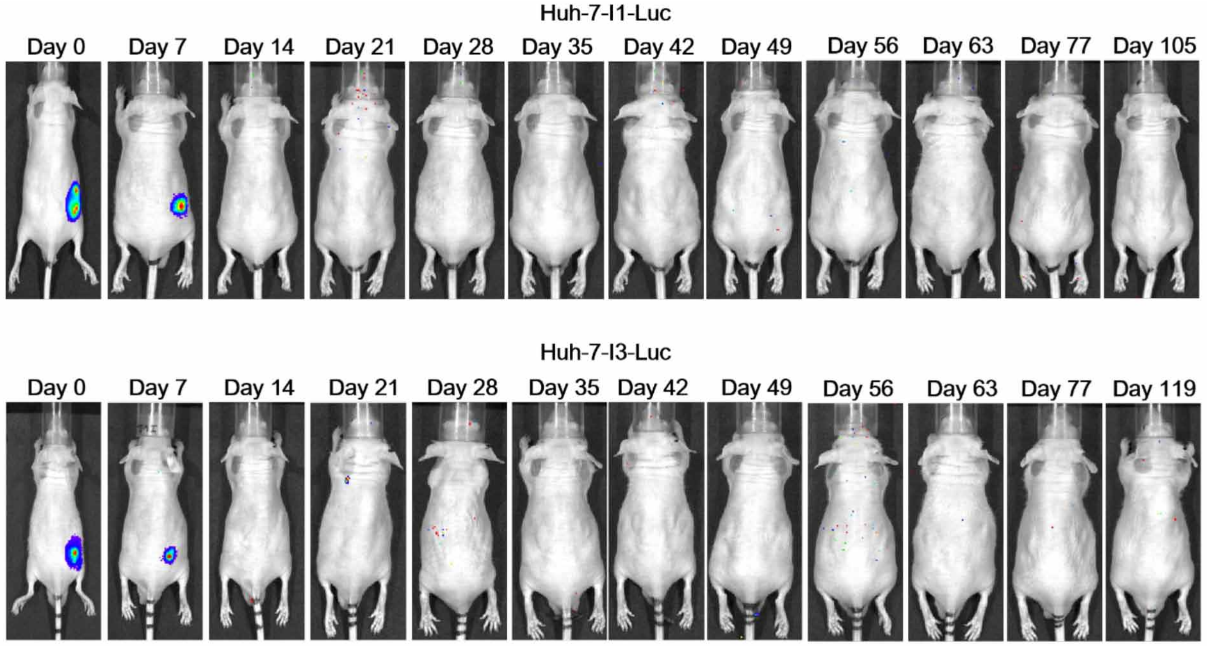

	Tumor incidence
Huh-7-I1	0/2
Huh-7-I3	0/2

Supplementary Figure S4: Highly invasive Huh-7 cells (Huh-7-I1 and Huh-7-I3) expressing luciferase were established by transfection with luciferase, followed by selection with G418 for four weeks. Selected cells were injected into nude mice and the tumor incidence was examined.

Supplementary Table S1: Oligonucleotide sequences for RT-PCR

Gene	Primer Sequences
Cyclin A	F 5′-TGAACTACATTGATAGGTTCCTGT-3′<br>Rv 5′-TGACTGTTGTGCATGCTGTGGTGC-3′
Cyclin D	Fw 5′-GCCAACCTCCTCAACGACCGG-3′<br>Rv 5′-GTCCATGTTCTGCTGGGCCTG-3′
Cyclin E	Fw 5′-ATAATGCAGTCTGTGCAGAC-3′<br>Rv 5′-GTTGTGTGCATCTTCATCAG-3′
AKR1B10	Fw 5′-AGCTTGGCTAAAATGGGACA-3′<br>Rv 5′-AACGTGTTGCAATCCTCTCA-3′

Supplementary Table S2: Oligonucleotide sequences for siRNAs

Accession no.	Gene name	siRNA sequence
NM_006761.4	YWHAE (14-3-3ε)	F: 5′-AAACCAUUACAACGAAGUCCCUCCC-3′<br>R: 5′-GGGAGGGACUUCGUUGUAAUGGUUU-3′
NM_020299.4	AKR1B10	sequence 1
		F: 5′-GCUCUUGAACAAACCUGGACUGAAA-3′<br>R: 5′-UUUCAGUCCAGGUUUGUUCAAGAGC-3′
		sequence 2
		F: 5′-GCCCAGGUUCUGAUCCGUUUCCAUA-3′<br>R: 5′-UAUGGAAACGGAUCAGAACCUGGGC-3′
NM_001904.3	CTNNB1 (β-catenin)	F: 5′-CCACAGCUCCUUCUCUGAGUGGUAA-3′<br>R: 5′-GGUGCUAUCUGUCUGCUCUAGUAAU-3′

Supplementary Table S3: 14-3-3ε/β-catenin increased genes in hepatocellular carcinoma

Gene symbol	Accession no.	Fold changes	
		14-3-3ε vs. Control	14-3-3ε/β-catenin siRNA vs. 14-3-3ε
BMPR2	NM_001204	2.08927	−2.25281
SNORD58A	NR_002571	2.03351	−2.46088
MAP2	NM_002374	2.64114	−3.05109
CHI3L1	NM_001276	3.47411	−2.34915
MATN3	NM_002381	2.99135	−2.38953
AKR1C3	NM_003739	5.75737	−2.00836
IL18	NM_001562	19.7617	−8.58105
AKR1B10	NM_020299	5.38145	−13.1227
SLC7A7	NM_003982	2.40097	−2.65859
PTGR1	NM_001146108	2.2573	−3.78931
SPARC	NM_003118	3.80172	−2.2331
PIP4K2A	NM_005028	2.05739	−2.12107
NFE2L2	NM_006164	2.29361	−2.25597
RGL1	NM_015149	3.41722	−2.2166
LMLN	NM_001136049	2.22254	−3.79571
ANKRD1	NM_014391	32.5929	−2.44794
IFFO2	NM_001136265	2.61125	−2.15042
MAL2	NM_052886	2.11264	−3.14979
NOX1	NM_007052	3.82203	−2.22099
JAG1	NM_000214	2.18321	−2.12992
LOC100134868	NR_004846	3.53102	−2.65705
CXCL5	NM_002994	4.25201	−2.05789
BCAS1	NM_003657	4.86668	−8.11185
CYP24A1	NM_000782	6.1167	−2.40276
GBP2	NM_004120	2.00719	−2.01949
PDLIM3	NM_014476	2.12958	−2.00331
KRT18	NM_000224	2.08964	−2.23643
TAGLN2	NM_003564	2.38661	−2.00397
LASS6	NM_203463	7.30736	−2.24522
PCSK5	NM_006200	2.50404	−2.12461
ANXA3	NM_005139	20.2831	−4.26316
SS18	NM_001007559	2.18654	−2.7797
VIM	NM_003380	12.0352	2.27E-13

Supplementary Table S4: 14-3-3ε/β-catenin decreased genes in hepatocellular carcinoma

Gene symbol	Accession no.	Fold changes	
		14-3-3ε vs. Control	14-3-3ε/β-catenin siRNA vs. 14-3-3ε
NR6A1	NM_033334	−2.28932	2.08922
LOC401357	AK125790	−3.08758	2.82648
LOC646870	AK130460	−2.39141	2.64155
